# Robust Hund rule without Coulomb repulsion and exclusion principle in quantum antiferromagnetic chains of composite half spins

**DOI:** 10.1088/1361-648X/aae169

**Published:** 2018-09-14

**Authors:** Solomon F Duki, Yi-Kuo Yu

**Affiliations:** National Center for Biotechnology Information, National Library of Medicine, National Institutes of Health, Bethesda, MD 20894, United States of America

**Keywords:** spin chain, antiferromagnetism, Hund rule

## Abstract

Quantum spin chains with composite spins have been used to approximate conventional chains with higher spins. For instance, a spin 1 (or $\frac{3}{2}$) chain was sometimes approximated by a chain with two (or three) spin $\frac{1}{2}$’s per site. However, little examination has been given as to whether this approximation, effectively assuming the first Hund rule per site, is valid and why. In this paper, the validity of this approximation is investigated numerically. We diagonalize the Hamiltonians of spin chains with a spin 1 and $\frac{3}{2}$ per site and with two and three spin $\frac{1}{2}$’s per site. The low energy excitation spectrum for the spin chain with *M* spin $\frac{1}{2}$’s per site is found to coincide with that of the corresponding conventional chain with one spin $\frac{M}{2}$ per site. In particular, we find that as the system size increases, an increasingly larger block of consecutive lowest energy states with maximal spin per site is observed, robustly supporting the first Hund rule even though the exclusion principle does not apply and the system does not possess Coulomb repulsion. As for why this approximation works, we show that this effective Hund rule emerges as a plausible consequence when applying to composite spin systems the Lieb–Mattis theorem, which is originally for the ground state of ferrimagnetic and antiferromagnetic spin systems.

## 1. Introduction

Quantum spin chain models are suitable for studying interacting quantum many-body systems with strong electron correlations, broken translational symmetry, and quantum fluctuations. One physical problem that is best studied in such models is magnetism [1, 2]; another example of growing interest in using spin chain models is the study of quantum information transfer and quantum information processing, the core parts of quantum information theory [3].

Typically a conventional Heisenberg spin chain is described by the Hamiltonian

(1) $$H=\frac{1}{2}\sum_{i\neq j}J_{ij}{\vec{S}}_{i}\cdot {\vec{S}}_{j}$$

where ${\vec{S}}_{i}$ is the spin-s operator at the *i* th site, positive (negative) *J_ij_* denotes antiferromagnetic (ferromagnetic) coupling between spins at sites *i* and *j*, and the coupling strength ∣*J_ij_*∣ generally depends on the separation distance ∣*i* – *j*∣. In this study, we assume *J_ij_* = *J* > 0 if *i* and *j* are nearest neighbors and 0 otherwise.

For the antiferromagnetic nearest neighbor coupling that we assume, the infinite length limit of the spin $\frac{1}{2}$ chain is exactly solvable via the Bethe ansatz [4] or by using the quantum inverse method [5]. However, for chains of higher spins the exact solution is unattainable in the infinite size limit. Very extensive studies have been done both theoretically and experimentally since Haldane [6–8] pointed out the drastic differences between integer and half-integer spin chains. In the infinite length limit, the energy difference (gap) between the first excited state and the ground state remains finite for an integer spin chain but vanishes for a half-integer spin chain.

For finite size spin-chains, a fairly large number of studies have also been done to investigate different aspects of magnetic materials. These include the fundamental origins of magnetism in metallic ions [9], impurity effects on chains [10], boundary effects [11], and the effect of even-odd number of sites on the eigenpairs of the chain [12].

In a somewhat different context, there have been earlier studies to approximate a spin 1 (or $\frac{3}{2}$) chain by a spin-chain with two (or three) spin $\frac{1}{2}$’s per site to study different aspects of finite size antiferromagnetic clusters [13–16]. However, little examination has been given as to whether this approximation, effectively assuming the first Hund rule per site, is valid and why should it work except that replacing one spin $\frac{M}{2}$ by *M* spin $\frac{1}{2}$’s maps the Hamiltonian into a solvable portion (made of spin $\frac{1}{2}$ chains) and interaction portions that some authors [15, 16] treated perturbatively. Evidently, if the multiple spin $\frac{1}{2}$’s on the same site interact strongly in a ferromagnetic manner, the spins on the same site would tend to line up and the first Hund rule emerges effectively. What is fascinating, however, is that the first Hund rule persists without any on-site ferromagnetic interaction, not to mention that the spin chains possess neither exclusion principle nor Coulomb repulsion, which are the foundation for the first Hund rule in atoms with multiple electrons. In this paper we investigate numerically the robustness of the first Hund rule in spin chains using chains of even number of sites with both periodic and open boundary condition. In addition to showing numerically that the first Hund rule is more pronounced in the periodic chain than in the open chain, we also provide an explanation for the first Hund rule based on the Lieb–Mattis theorem [17].

## 2. The model

The one-dimensional model we study here originates from [15]. It has *N* sites, and on each site there are *M* spin $\frac{1}{2}$ written as $\{{\vec{S}}_{\lambda }{\}}_{\lambda =1}^{M}$. The spin $\frac{1}{2}$’s on the same site do not interact with one another but each interacts antiferromagnetically with $\frac{1}{2}$ spins on the nearest neighboring sites. That is, the Hamiltonian is of the same form as (1) except that ${\vec{S}}_{i}$, the spin on site *i*, now represents

(2) $${\vec{S}}_{i}\Rightarrow {\vec{S}}_{i}(M)=\sum_{\lambda =1}^{M}{\vec{S}}_{\lambda ,i}$$

with *λ* being the index for spin $\frac{1}{2}$’s on a given site. The Hilbert space per site is constructed by taking the tensor product of $MC^{2}$, the Hilbert space of a spin $\frac{1}{2}$. Hence the Hilbert per site can be written in short as $(C^{2})\;{}^{⊗M}$. The component of the spin vector ${\vec{S}}_{\lambda }$ (1 ⩽ λ ⩽ *M* on each site) can be written as

(3) $$S_{\lambda }^{a}\equiv \overset{\text{with}\;(\lambda -1)I_{2}s}{\overset{︷}{I_{2}⊗I_{2}⊗\cdots }}⊗\frac{\sigma_{a}}{2}\overset{\text{with}\;(M-\lambda )I_{2}s}{\overset{︷}{⊗I_{2}⊗I_{2}⊗\cdots }}$$

where *I*_2_ is the identity matrix of rank 2 and *σ*_*a*=*x,y,z*_ are the two dimensional Pauli matrices.

The spin operator at the *i*th site acts on the full Hilbert space but non-trivially only on site *i*. In short we write the *i*th spin as

(4) $$S_{i}^{a}\equiv \overset{\text{with}\;(i-1)I_{2^{M}}s}{\overset{︷}{I_{2^{M}}⊗I_{2^{M}}⊗\cdots }}⊗S_{i}^{a}(M)⊗\overset{\text{with}\;(N-i)I_{2^{M}}s}{\overset{︷}{I_{2^{M}}⊗I_{2^{M}}⊗\cdots }}$$

where *I*_2^*M*^_ is the identity matrix (in 2^*M*^ dimension) and *S_i_*(*M*) is the spin operator obtained by taking the tensor product of *M* spin $\frac{1}{2}$ operators, as shown in equations (2) and (3), at the *i*th site.

Since the Hamiltonian is the sum of the products of spin operators of neighboring sites, *H* grows exponentially with both *M* and *N*; i.e. *H* ~ *D*^*N*^ × *D*^*N*^ where *D* = *2*^*M*^ is the dimension of the Hilbert space per site from *M* spin $\frac{1}{2}$’s. However, similar to (1), the resulting Hamiltonian commutes with the magnetization of the system $M=S_{\text{tot}}^{z}\equiv \sum_{i=1}^{N}S_{i}^{z}=\sum_{i=1}^{N}(\sum_{\lambda =1}^{M}S_{\lambda ,i}^{z})$. Consequently, The Hamiltonian can be written in a block diagonal form, with each block having a fixed M. In addition, the Hamiltonian is invariant under rotation that mixes the states with $S_{\text{tot}}^{z}=-S,-S+1,\ldots ,S$. That is, all eigenstates with the same *S*_tot_ = *S* but with different $S_{\text{tot}}^{z}$ have identical energies. Evidently, the sector with the minimum absolute value of $S_{\text{tot}}^{z}$ contains all the energy levels of the system. Therefore the task of finding eigenpairs is significantly simplified by diagonalizing the subspace with a particular $S_{\text{tot}}^{z}=M$ at a time. Henceforth we refer to this subspace as the M subspace.

## 3. *M* spin $\frac{1}{2}$ versus one spin $\frac{M}{2}$ per site: numerical studies

To compare the low energy excitations of these two systems, we diagonalized both Hamiltonians and obtained at least the first 150 eigenpairs for *M* = 2 and 3 for different chain lengths. Many eigenpairs are needed in order to identify the level up to which these two systems are equivalent. Although the Lanczos [18, 19] algorithm is routinely used to calculate the first few eigenvalues of the Heisenberg Hamiltonian, it is not the most efficient when one is interested in getting a large number of lowest eigenvalues [19]. Our numerical solutions are obtained using the Anasazi [20] package of the Trilinos [21, 22] project, an objected oriented framework that integrates several algorithms for large scale physical problems. Trilinos efficiently enables users to have combined access to eigensolvers with effective preconditioners, sparse-solvers and partitioning methods.

For diagonalization, we used the generalized Davidson’s algorithm [23–25] as our eigensolver along with Ifpack [26], the incomplete factorization preconditioning package having a large selection of algebraic preconditioning. The generalized Davidson’s algorithm has advantages over many of the Anasazi solvers as its computational cost is lower for calculating a fairly large number of eigenpairs. This is due to the fact that the storage of the factors, needed for a rapid sparse factorization process, consumes more and more memory as the system size increases. All eigenpairs are calculated with a convergence tolerance and precondition drop tolerance of 10^−10^.

For chains with *M* =2 or 3 spin $\frac{1}{2}$’s per site, based on $|\frac{1}{2}\rangle ⊗|\frac{1}{2}\rangle =|1\rangle ⊕|0\rangle$ and $|\frac{1}{2}\rangle ⊗|\frac{1}{2}\rangle ⊗|\frac{1}{2}\rangle =|\frac{3}{2}\rangle ⊕2|\frac{1}{2}\rangle$, the total Hamiltonian can therefore be decomposed as

(5) $$H=\sum_{\gamma =0}^{M}h_{\gamma }$$

where *h*_*γ*_ is the term that has *γ* number of sites with non-maximal spins (spin 0 sites for *M* = 2 and spin $\frac{1}{2}$ sites for *M* = 3). We have numerically observed that in (5) the terms change into having fewer non-maximal spin sites also have lower energies.

That is, these spin 0 and $\frac{1}{2}$ sites can be considered effectively as impurities that increase the energies of the system. Since the lowest energy states comes from the first term in (5), this means that only the high spin state at each site contributes to the lower energy eigenstates. This is the first Hund rule that is observed in atomic and molecular systems except that we do not have Coulomb repulsion or exclusion principle.

Table 1 shows the low energy spectra of periodic chains with two spin $\frac{1}{2}$ per site. The degeneracy (*D*) of each energy level is also provided. In this table, cells colored in orange are states having exact correspondence in a conventional chain (with one spin 1 per site) of the same size; energies in cells colored in blue are from chains each with one impurity site. An impurity (spin 0) site effectively makes a chain of one site less with open boundary condition. For example, the first blue state in the 8-site column site is exactly the triplet ground state of an open 7-site spin 1 chain, which also represents an 8-site chain with an impurity site. Because of the translational symmetry of the system, the impurity could open the periodic chain at any of the 8 sites, resulting the 24 fold degeneracy shown in the table. In a similar fashion, if we have *γ* ⩾ 2 impurities in the system, the spin chain can be effectively opened up at up to *γ* number of sites. Cells containing energies corresponding to such states are colored in yellow.

In table 1, one may also enumerate *n*_*s*_, the number of states lie below the lowest energy impurity state. It is clear that *n_s_* increases as the chain size increases. These numbers are shown in table 2. The fact that *n_s_* increases with size indicates that in the low energy sector of a long chain, the two spin $\frac{1}{2}$’s are aligned to get the maximum spin of *s* = 1 at each site, supporting the first Hund rule.

To investigate the effect of boundary conditions, we have also analyzed chains of *N* = 8, 10, 12, 14 sites with two spin $\frac{1}{2}$’s per site but with open boundary condition. The results are summarized in table 3. In this case, since the spins at site 1 and site N can no longer contribute a negative energy due to the absence of coupling, the ground state energy will appear higher in comparison with the corresponding periodic chain. This can be easily seen by comparing the ground state energies in table 3 with those in table 1. One important thing to note is that when there is one impurity, one expects that having the impurity on one of the edge sites is energetically more favorable. This is because an impurity site in the middle of the chain interrupts two couplings while an impurity on the edge only interrupts one. Another interesting point to note is that when the single impurity is placed on the edge of the chain, it effectively reduces the open chain size by one. The energies corresponding to states of one impurity site on the edge are placed in cells colored in blue. These energies are also found in table 1 except with different degeneracies *D*. For example, the first blue state in the 10-site column is exactly the triplet ground state of an open 9-site spin 1 chain, which also represents a 10-site open chain with an impurity site on the edge. Because the two edge sites are equivalent, the number of states corresponding to this energy becomes *D* = 2 × 3 = 6 as opposed to *D* = 10 × 3 = 30 for the periodic chain. For the open chains, we have also identified states corresponding to one impurity sitting at an interior site; the corresponding energies are displayed in cells colored in cyan. For states corresponding to two or more impurities, we again display their energies in cells colored in yellow.

In table 3, one may again enumerate *n_s_*, the number of states lie below the lowest energy impurity state. One still observes that *n_s_* increases as the chain size increases. These *n_s_* numbers are shown in table 4. It has not escaped our attention that for open chains *n_s_* does not grow with the system size as fast as for periodic chains. This can be understood as follows: for the same chain length the ground state energy of an open chain is higher than the periodic chain while the lowest energy impurity states have the same energies for both periodic and open chains. That is, the energy difference between the lowest energy state and the lowest energy impurity state is larger for the periodic chains, hence able to accommodate more impurity-free states before encountering the lowest energy impurity state. Nevertheless, because *n_s_* increases with size still, in the low energy sector of a long chain, the two spin $\frac{1}{2}$’s are aligned to get the maximum spin of *s* = 1 at each site, agreeing with the first Hund rule.

We have also obtained a similar result when we put three spin $\frac{1}{2}$ per site and compared its energy spectrum with that of the conventional one spin $\frac{3}{2}$ per site of the same chain length. Because the Hilbert space per site has increased from 4 to 8 in this case, we only show results for chains, be they open or periodic, with 6 sites and 8 sites. Tables 5 and 6 display respectively the results for periodic and open chains.

The three spin $\frac{1}{2}$’s per site model has more degrees of freedom at each site than that of one spin $\frac{3}{2}$ per site. In terms of the irreducible representations we have $|\frac{1}{2}\rangle ⊗|\frac{1}{2}\rangle ⊗|\frac{1}{2}\rangle =|\frac{3}{2}\rangle ⊕2|\frac{1}{2}\rangle$. So there are two spin $\frac{1}{2}$ sectors that emerge and they turn out to have higher energies. (A spin $\frac{1}{2}$ site in this case thus can be viewed as an impurity site.) As a result, the lower energy states of the three spin $\frac{1}{2}$ per site chain correspond to those where all the $\frac{1}{2}$ spins at each site are aligned to maximize the spin per site. By comparing the 6 site results with the 8 site results, we find that the number of lower energy states with maximal spin per site increases as the size increases regardless of the boundary condition, agreeing with the first Hund rule.

## 4. *M* spin $\frac{1}{2}$ versus one spin $\frac{M}{2}$ per site: analytic understanding via a twist of Lieb–Mattis theorem

In this section, we will first review the Lieb–Mattis theorem and then use it in conjunction with the smallness of the Haldane gap (when compared to the coupling *J*) to elucidate the observed Hund rule in spin chains containing multiple spin $\frac{1}{2}$ per site.

Lieb and Mattis [17] considered a generic Heisenberg spin system, similar to (1), within which two sublattices *A* and *B* can be identified as follows: the spin coupling among spins within the same sublattice is smaller than *g*^2^ (*J*_*i*(*A*),*j*(*A*)_ ⩽ *g*^2^ and *J*_*i*(*B*),*j*(*B*)_ ⩽ *g*^2^) while the coupling between spins in different sublattices is larger than *g*^2^ (*J*_*i*(*A*),*j*(*B*)_ ⩾ *g*^2^). They worked out the case for *g*^2^ = 0 and then extend it to general *g*^2^ values. For our purpose, however, we only need to consider and review the *g*^2^ = 0 case. Let us also note that for open boundary condition the even numbered sites and the odd numbered sites constitute the two sublattices needed. Even though an open chain is allowed to have an odd number of total sites, for a periodic chain, the total number of sites must be even in order to have two clearly defined sublattices. It is for this particular reason that all tables, shown in the previous section, are for even number of sites.

One of the key steps involves identifying two operators ${\vec{S}}_{\text{tot}}^{2}\equiv {\left(\sum_{i}{\vec{S}}_{i}\right)}^{2}$ and $S_{\text{tot}}^{z}$, with respective eigenvalues *S*(*S* + 1) and −S⩽M⩽S, that commute with each other and with the Hamiltonian (1). These commutation relations, elaborated in some detail in appendix B, ensure that the eigenstates can be made simultaneously diagonal in *H*, ${\vec{S}}_{\text{tot}}^{2}$ and $S_{\text{tot}}^{z}$. This means that each eigenstate can be assigned a total spin *S* and its *z* component $S_{\text{tot}}^{z}=M$. Because −S⩽M⩽S, there are 2*S* + 1 states corresponding to a given total spin *S*. Note that each term ${\vec{S}}_{i}\cdot {\vec{S}}_{j}$ of *H* is rotationally invariant, hence so is *H*. Applying a rotational operator to an eigenstate of $S_{\text{tot}}^{z}=M$ (and total spin *S*) produces, without energy change, a new state that is a linear combination of the (2*S +* 1) states of different $S_{\text{tot}}^{z}$ (but of the same total spin *S*). Given that the rotation introduced can be arbitrary, all these 2*S* + 1 states (with different M) must have the same energy. This means that every energy eigenvalue has a corresponding eigenfunction (representative) in the smallest ∣M∣ subspace (M=0 subspace if *S* is an integer).

The Lieb–Mattis theorem states that the ground state of *H* belongs to total spin S=s with $s\equiv |S_{A}-S_{B}|$ and *S*_*A*_ (*S*_*B*_) is the maximum possible spin ∑_*i*(*A*)_ *S*_*i*(*A*)_ (∑_*i*(*B*)_ *S*_*i*(*B*)_) on the A (B) sublattice. The theorem relies on the results (when *g*^2^ = 0) *E*(*S* + 1) > *E*(*S*) for all S⩾s and E(S)>E(s) for all S<s, where *E*(*S*) denotes the lowest energy eigenvalue belonging to total spin *S*. The key idea is to show that in a $S_{\text{tot}}^{z}=M$ subspace the lowest energy state of *H* is not orthogonal to the lowest energy state of a Hamiltonian $H^{\prime }=({\vec{S}}_{\text{tot}}{)}^{2}-{\vec{S}}_{A}^{2}-{\vec{S}}_{B}^{2}$ where ${\vec{S}}_{A}\equiv \sum_{i(A)}{\vec{S}}_{i(A)}$, ${\vec{S}}_{B}\equiv \sum_{i(B)}{\vec{S}}_{i(B)}$ and ${\vec{S}}_{\text{tot}}=\sum_{i}{\vec{S}}_{i}={\vec{S}}_{A}+{\vec{S}}_{B}$. Obviously, the lowest energy state of the latter has maximized ${\vec{S}}_{A}^{2}$ and ${\vec{S}}_{B}^{2}$ but minimized ${\vec{S}}_{\text{tot}}^{2}=M(M+1)$ (evidently M cannot be smaller than s). Therefore, in the M subspace (with $S_{\text{tot}}^{z}=M$), the lowest energy state of *H* must have $S_{\text{tot}}=M$ (with M⩾s) as well. (For if the lowest energy state of *H* correspond to a different $S_{\text{tot}}\neq M$, the corresponding wave function must be orthogonal to any wave function with $S_{\text{tot}}=M$; hence zero overlap with the lowest energy state wave function of *H*′.)

Relevant derivations for the aforementioned results will be provided in appendix B. Here we only highlight the key points. Basically, for the generic Hamiltonian of Lieb and Mattis, the lowest energy state in a $S_{\text{tot}}^{z}=M$ subspace is a linear combination of all basis states (under a specific convention) yielding $S_{\text{tot}}^{z}=M$ with positive coefficients only. For the soluble Hamiltonian *H*′, the lowest energy state in the subspace $S_{\text{tot}}^{z}=M$ also has this property, i.e. it is a linear combination of all basis states yielding $S_{\text{tot}}^{z}=M$ with positive coefficients. However, we can look at *H*′ from a different perspective. Given that *H*′ can be written as $({\vec{S}}_{\text{tot}}{)}^{2}-{\vec{S}}_{A}^{2}-{\vec{S}}_{B}^{2}$, we know its lowest energy state has maximum *S*_*A*_ and *S*_*B*_ but with $S_{\text{tot}}=s=|S_{A}-S_{B}|$. In the context of the *M* spin $\frac{1}{2}$ per site model, this means that all spin $\frac{1}{2}$ on the same site must line up to form maximum spin in order to have lowest energy in *H*′. That is, the basis set contributing to the lowest energy state of *H*′ must have spin $\frac{M}{2}$ at each site. Since the basis set contributing to the lowest energy state of *H*′ and that of *H* are the same, the lowest energy state of *H* in the same subspace must be comprised of spin $\frac{M}{2}$ at each site. This is the origin of the Hund rule in the ground state of the composite spin chain studied here. One must note that even though the ground state of *H* is effectively a spin $\frac{M}{2}$ chain, the robustness of this Hund rule requires the Haldane gap to be small. Imagine that the energy difference between the ground state of the spin $\frac{M}{2}$ chain and the ground state of a spin $\frac{M}{2}$ chain with impurity (with some sites having spin less than $\frac{M}{2}$) is smaller than the Haldane gap, then the excitations of *H* (with multiple spin $\frac{1}{2}$ per site) are no longer describable by the spin $\frac{M}{2}$ chain.

## 5. Summary and conclusion

In summary, we considered an antiferromagnetic chain of *M* spin $\frac{1}{2}$’s per site to gain a better understanding of the relations between this composite spin chain and a spin $\frac{M}{2}$ chain. For chains of finite lengths, we solved the Hamiltonian using exact diagonalization and obtained the eigenpairs of the lowest energies for chains with two and three spin $\frac{1}{2}$’s per site. To make a comparison, we have also diagonalized the Hamiltonian of the conventional spin 1 and spin $\frac{3}{2}$ chains of the same sizes. We find that for the *M* spin $\frac{1}{2}$ per site chain, as the system size increases, its excitation spectrum coincides better with that of the conventional spin $\frac{M}{2}$ chain. That is, the system displays the first Hund rule robustly despite that the spin chain with *M* spin $\frac{1}{2}$’s per site lacks Coulomb repulsion and exclusion principle, the mechanism for the first Hund rule in atomic and molecular systems. We elucidate this robust Hund rule in spin chains by using the Lieb–Mattis theorem and the smallness of the Haldane gap.

Although the emergence of the first Hund rule in composite spin chains can be viewed as consequences of the Lieb–Mattis theorem, their connections to the Lieb–Mattis theorem are nontrivial. This is evidenced by the fact that the first Hund rule in composite spin chains has been implicitly assumed/used for decades, no reference was made to the Lieb–Mattis theorem.

## Figures and Tables

**Table 1.** The energy eigenvalues of periodic chains with two spin $\frac{1}{2}$ per site. Size is the length of the chain and *D* records the total number of (degenerate) states for the given energy level. Each state shown in orange has a corresponding state in the conventional spin 1 chain. States shown in blue each contains one impurity (spin 0) site. States containing two or more impurity sites are colored in yellow. It is worth noting that an impurity site breaks the periodic chain, making it effectively an open chain with one site less. Because it makes no difference at which site of the periodic chain a single impurity sits, the degeneracies *D* for energy levels from states containing one impurity are always multiples of the size of the chain, as shown by the *D* values in the blue cells.

Size 8	*D*	Size 10	*D*	Size 12	*D*	Size 14	*D*
−11.337	1	−14.0941	1	−16.8696	1	−19.6551	1
−10.7434	3	−13.5693	3	−16.3854	3	−19.1962	3
−9.59656	5	−12.5971	5	−15.5294	5	−18.4532	6
−9.3436	6	−12.4585	6	−15.4858	6	−18.4227	5
−9.21845	6	−12.2661	6	−15.2458	6	−18.1792	6
−8.65154	1	−11.8457	1	−14.9888	1	−18.0213	1
−8.63453	24	−11.718	1	−14.8058	3	−17.815	3
−8.55499	6	−11.7145	3	−14.7064	1	−17.6462	1
−8.5085	1	−11.5065	6	−14.5497	6	−17.5829	6
−8.4849	3	−11.4329	30	−14.5061	10	−17.5477	10
−8.30358	8	−11.4315	6	−14.4083	6	−17.3907	6
−8.14573	10	−11.3843	10	−14.3359	10	−17.3411	10
−8.06951	10	−11.2588	10	−14.2781	7	−17.3381	2
−7.88305	2	−11.2202	10	−14.2665	2	−17.3097	7
−7.87562	7	−11.1548	7	−14.2304	36	−17.3008	10
−7.70385	10	−11.1187	2	−14.192	10	−17.1879	6
−7.686	2	−10.9912	6	−14.1375	6	−17.0304	6
−7.68595	6	−10.9784	10	−14.1343	6	−17.0283	42
−7.52385	40	−10.8033	2	−14.0886	12	−17.0042	2
−7.50231	24	−10.74	10	−13.8803	2	−16.9694	6
−7.48164	10	−10.6393	6	−13.8498	6	−16.9591	6
−7.37027	8	−10.5871	3	−13.841	10	−16.9316	14
		−10.5706	10	−13.8324	2	−16.9082	3
				−13.7986	3	−16.9081	2
						−16.8984	10

**Table 2.** The number of impurity free states (*n*_*s*_) with energies lower than that of any impurity state. Periodic spin chains with two spin $\frac{1}{2}$ per site are considered. Specifically, *n*_*s*_ sums the *D* values in the orange cells in table 1 from the top till encountering the first blue cell. As the chain length increases, a larger consecutive block of lowest energy states from a periodic chain with two spin $\frac{1}{2}$ per site can be mapped bijectively to a consecutive block of lowest energy states of a conventional periodic chain with one spin 1 per site.

Size	8	10	12	14
*n_s_*	22	32	67	89

**Table 3.** The energy eigenvalues of open chains with two spin $\frac{1}{2}$ per site. Size is the length of the chain and *D* records the total number of (degenerate) states for the given energy level. Each state shown in orange has a corresponding state in the conventional spin 1 chain. States shown in blue each contains one impurity (spin 0) sitting on one of the edge sites. The energies of states corresponding to one impurity sitting at an interior site are displayed in cells colored in cyan. States containing two or more impurity sites are colored in yellow.

Size 8	*D*	Size 10	*D*	Size 12	*D*	Size 14	*D*
−10.12460	1	−12.8946	1	−15.674	1	−18.4599	1
−9.92276	3	−12.7562	3	−15.5769	3	−18.3907	3
−9.04914	5	−11.9907	5	−14.8851	5	−17.7519	5
−8.89788	3	−11.8691	3	−14.7872	3	−17.6723	3
−8.80438	3	−11.8227	3	−14.7665	3	−17.666	3
−8.63453	6	−11.6437	1	−14.6162	1	−17.5417	1
−8.59709	1	−11.4329	6	−14.3716	5	−17.3149	5
−8.30358	2	−11.3773	5	−14.2543	3	−17.2067	3
−8.30157	5	−11.2569	3	−14.232	3	−17.2048	3
−8.19805	3	−11.2202	2	−14.2304	6	−17.0717	1
−8.08297	3	−11.2	3	−14.0886	2	−17.0283	6
−7.91838	1	−11.0435	1	−14.0859	1	−16.9316	2
−7.83021	6	−10.865	5	−13.8839	5	−16.871	5
−7.76298	5	−10.807	3	−13.8617	5	−16.8364	5
−7.70367	3	−10.801	5	−13.837	3	−16.8053	3
−7.67724	3	−10.7566	3	−13.7841	3	−16.775	3
−7.65059	5	−10.7178	7	−13.7711	7	−16.7661	3
−7.64575	6	−10.684	3	−13.75	3	−16.7548	7
−7.57210	3	−10.6353	3	−13.7016	3	−16.6968	3
−7.55097	7	−10.6345	6	−13.6677	1	−16.6784	1
−7.52385	10	−10.5898	1	−13.6217	5	−16.6428	1
−7.50231	6	−10.539	5	−13.6173	1	−16.6295	5
−7.45629	3	−10.5208	1	−13.5283	5	−16.5619	5
−7.43039	1	−10.5111	10	−13.4541	5	−16.4693	5
−7.37027	6	−10.4854	6	−13.4329	6	−16.4611	3
−7.37027	3	−10.476	6	−13.4314	10	−16.4208	5
−7.33789	5	−10.4148	5	−13.4206	3	−16.4023	3
−7.31928	5	−10.4087	3	−13.4056	6	−16.3926	3
−7.28357	2	−10.3957	5	−13.4017	3	−16.3829	3
−7.28182	1	−10.3703	6	−13.3908	5	−16.3827	1
−7.18870	2	−10.324	3	−13.3722	3	−16.3411	3
−7.15232	5	−10.3036	2	−13.3608	3	−16.314	10
−7.13658	18	−10.2919	5	−13.329	1	−16.3114	7
−7.11363	6	−10.2833	3	−13.2819	1	−16.3038	1
−7.08105	3	−10.2811	3	−13.2803	6	−16.2897	6
−7.06249	18	−10.2488	2	−13.2631	7	−16.2825	3
−7.06249	9	−10.2159	6	−13.2591	3	−16.2304	6
		−10.2075	1	−13.2412	3	−16.2291	1
		−10.1894	1	−13.2261	2	−16.188	5
		−10.1313	7	−13.2202	2	−16.1514	2
				−13.2141	6	−16.1509	6

**Table 4.** The number of impurity free states (*n*_*s*_) with energies lower than that of any impurity state. Open spin chains with two spin $\frac{1}{2}$ per site are considered. Specifically, *n*_*s*_ sums the *D* values in the orange cells, in table 3), from the top till encountering the first blue cell. As the chain length increases, a larger consecutive block of lowest energy states from a periodic chain with two spin $\frac{1}{2}$ per site can be mapped bijectively to a consecutive block of lowest energy states of a conventional open chain with one spin 1 per site.

Size	8	10	12	14
*^n^s*	15	16	27	28

**Table 5.** The energy eigenvalues of periodic chains with three spin $\frac{1}{2}$ per site. Size is the length of the chain and D records the total number of (degenerate) states for the given energy level. Each state shown in orange has a corresponding state in the conventional spin $\frac{3}{2}$ chain. States shown in blue each contains one impurity (spin $\frac{1}{2}$) site. States containing two or more impurity sites are colored in yellow. Because there are impurity sectors (two spin $\frac{1}{2}$ species) and it makes no difference at which site of the periodic chain a single impurity sits, the degeneracies *D* for energy levels from states containing one impurity are always multiples of twice the size of the chain, as shown by the *D* values in the blue cells.

Size 6	*D*	Size 8	*D*
−17.3928	1	−22.93	1
−16.6874	3	−22.3714	3
−15.2768	5	−21.2564	5
−14.0448	6	−20.2199	6
−14.0149	6	−20.1774	6
−13.6051	36	−19.5966	7
−13.1652	7	−19.2323	48
−12.6931	10	−19.1791	6
−12.6532	1	−19.1423	1
−12.5726	10	−19.1324	10
−12.2132	60	−18.9591	10
−12.1265	12	−18.4108	3
−11.8957	3	−18.2348	10
−11.7748	2	−18.2164	1
−11.5238	24	−18.1956	2
−11.4119	1	−18.1532	80
−11.3898	36	−18.0253	16

**Table 6.** The energy eigenvalues of open chains with three spin $\frac{1}{2}$ per site. Size is the length of the chain and D records the total number of (degenerate) states for the given energy level. Each state shown in orange has a corresponding state in the conventional spin $\frac{3}{2}$ chain. States shown in blue each contains one impurity (spin $\frac{1}{2}$) sitting on one of the edge sites. The energies of states corresponding to one impurity sitting at an interior site are displayed in cells colored in cyan. States containing two or more impurity sites are colored in yellow.

Size 6	*D*	Size 8	*D*
−14.7571	1	−20.3731	1
−14.4621	3	−20.1657	3
−13.6662	5	−19.5595	5
−12.8383	12	−18.8114	3
−12.7182	3	−18.7825	3
−12.6966	3	−18.5173	12
−12.11	7	−18.3098	5
−12.105	5	−18.2947	7
−11.7979	4	−18.0089	5
−11.7088	1	−17.9392	1
−11.7	5	−17.7732	4
−11.6457	20	−17.7372	3
−11.5766	3	−17.7167	3
−11.4869	3	−17.6352	3
−11.3055	12	−17.5296	20
−11.3023	4	−17.3042	5
−11.2242	3	−17.2035	12
−11.1161	12	−17.1978	7
−11.0684	5	−17.0331	3
−10.9248	12	−17.01	1
−10.7749	7	−17.0085	12
−10.6861	1	−16.999	5
−10.6629	5	−16.9789	3
−10.6349	20	−16.9436	4
−10.6093	12	−16.9357	5
−10.6053	20	−16.8604	5
−10.5362	20	−16.8562	1
−10.4873	8	−16.82	12

## Appendix A. Hilbert space decomposition

In this appendix we show how the Hilbert space of *M* spin $\frac{1}{2}$ per site gets decomposed into a direct sum of irreducible representations. Suppose that *V* is the Hilbert for a single spin $\frac{1}{2}$. When considering two spin $\frac{1}{2}$ on the same site, the direct product space *V* ⨂ *V* is described by a second order tensor which can be decomposed into the symmetric and antisymmetric parts. Thus if ${\hat{e}}_{n}$ (*n* = 1, 2) are bases that span *V*, then any second order tensor in the product space can be written in terms of the product bases that form the irreducible subspaces:

(A.1) $$\begin{array}{cc} {\hat{e}}_{n}⊗{\hat{e}}_{n^{\prime }} & =\frac{1}{2}({\hat{e}}_{n}⊗{\hat{e}}_{n^{\prime }}+{\hat{e}}_{n^{\prime }}⊗{\hat{e}}_{n})\\ & \;\;⊕\frac{1}{2}({\hat{e}}_{n}⊗{\hat{e}}_{n^{\prime }}-{\hat{e}}_{n^{\prime }}⊗{\hat{e}}_{n}). \end{array}$$

In terms of the Young diagram, these two subspaces are represented by  and  respectively, where  is the basic two dimensional representation.

For a site of *M* spin $\frac{1}{2}$, its Hilbert space has 2^*M*^ dimensions. The invariant subspaces can be obtained from the Young diagram, generalizing the decomposition procedure to *M* spin $\frac{1}{2}$. The Hilbert space at site *i* is a direct product *V*_*i*_ = *V* ⨂ *V* ⨂ *V* ⋯ ⨂ *V* (*M* times). Applying the Young diagram construction, the invariant sub spaces will be given as

This is the same as applying the Clebsch–Gordan formula repeatedly; i.e. if we represent each of the inequivalent irreducible subspaces of dimension *l* by *D*^(*l*)^, then

(A.2) $$D^{\left(l\right)}⊗D=D^{\left(l+1\right)}⊕D^{\left(l-1\right)}$$

where *D* represents the two dimensional representation for the single spin $\frac{1}{2}$ and *D*^(0)^ = *D*^(−1)^ = 0. The representations of the above Young diagrams can be obtained by repeated application of equation (A.2); i.e.

(A.3) $$\begin{array}{cc} V_{i} & =D^{⊗M}\equiv D⊗D⊗D⊗\cdots \\ & =\left(D^{\left(3\right)}⊕D^{\left(1\right)}\right)⊗D⊗D⊗\cdots \\ & =\left(D^{\left(4\right)}⊕D^{\left(2\right)}⊕D^{\left(2\right)}\right)⊗D⊗D⊗\cdots \\ & ={⊕}_{l=0}^{l=\lfloor \frac{M}{2}\rfloor }m_{l}D^{\left(M+1-2l\right)} \end{array}$$

where $\left\lfloor \frac{M}{2}\right\rfloor$ represents the largest integer that is smaller than or equal to $\frac{M}{2}$ and the *m_l_*’s are non-negative integers which represents the multiplicity of subspaces *D*^(*M*+1–2*l*)^, and they are given by

(A.4) $$m_{l}=\left\{\begin{array}{c} M\\ l \end{array}\right\}\equiv C_{l}^{M}-C_{l-1}^{M},$$

with $C_{l}^{M}=\frac{M!}{l!(M-l)!}$ and the understanding that $C_{-1}^{M}=0$. Note that a spin *s* representation has a dimension of 2*s* + 1, hence the representation of dimension *M* + 1 – 2*l* correspond to spin $\frac{M}{2}-l$. This allows us to write the total spin per site as a sum of operators acting on different invariant subspaces. Specifically,

(A.5) $${\vec{S}}_{i}=\underset{l=0}{\overset{l=\lfloor \frac{M}{2}\rfloor }{⊕}}m_{l}{\vec{S}}_{i}^{\left(\frac{M}{2}-l\right)}.$$

When a subspace, say *D*^(*M*+1–2*l*)^ is chosen for site *i*, the corresponding spin operator at that site must be of spin $\frac{M}{2}-l$.

The Hilbert space of the system of *N* sites is naturally written as

(A.6) $$\begin{array}{cc} V_{1} & ⊗V_{2}⊗\cdots ⊗V_{N}\\ & \;=\left({⊕}_{l=0}^{l=\lfloor \frac{M}{2}\rfloor }m_{l}D_{1}^{\left(M+1-2l\right)}\right)⊗\left({⊕}_{l=0}^{l=\lfloor \frac{M}{2}\rfloor }m_{l}D_{2}^{\left(M+1-2l\right)}\right)\\ & \;\;\;⊗\cdots ⊗\left({⊕}_{l=0}^{l=\lfloor \frac{M}{2}\rfloor }m_{l}D_{N}^{\left(M+1-2l\right)}\right). \end{array}$$

Obviously, the Hilbert subspace from the direct product of the *l* = 0 term within each *V_i_* is identical to the Hilbert space of a spin $\frac{M}{2}$ chain. When in *V_i_* a term with *l* ⩾ 1 is picked, the spin at site *i* in this Hilbert subspace becomes less than $\frac{M}{2}$, and we term the site an impurity site. Evidently, the final Hilbert space can be written as the direct sum of the Hilbert space of a spin $\frac{M}{2}$ chain and chains with impurities of various degrees located at different sites.

## Appendix B. Relevant details related to the Lieb–Mattis theorem

Let us start with the discussion of the commutation relations among the generic Hamiltonian (1), ${\vec{S}}_{\text{tot}}^{2}$ and $S_{\text{tot}}^{z}$. Let us begin with $\left[{\vec{S}}_{\text{tot}}^{2},S_{\text{tot}}^{z}\right]$. We first write

(B.1) $${\vec{S}}_{\text{tot}}^{2}=\sum_{l}{\vec{S}}_{l}^{2}+\sum_{l\neq l^{\prime }}{\vec{S}}_{l}\cdot {\vec{S}}_{l^{\prime }},$$

(B.2) $$S_{\text{tot}}^{z}=\sum_{i}S_{i}^{z}.$$

Evidently, $\left[{\vec{S}}_{l}^{2},S_{i}^{z}\right]$ when *i* ≠ *l*. When *i* = *l*, clearly $[(S_{i}^{z}{)}^{2},S_{i}^{z}]=0$ and it is easy to verify that $[(S_{i}^{x}{)}^{2}+(S_{i}^{y}{)}^{2},S_{i}^{z}]=0$ via angular momentum algebra. On the other hand, $[{\vec{S}}_{l}\cdot {\vec{S}}_{l^{\prime }},S_{i}^{z}]=0$ if *i* ≠ *l* and *i* = *l*′. When *i* = *l* or *l*′, the commutator is nonvanishing. However, if one were to compute $\left[{\vec{S}}_{l}\cdot {\vec{S}}_{l^{\prime }},S_{l}^{z}+S_{l^{\prime }}^{z}\right]$, one finds it equals zero. These relations guarantee that $[{\vec{S}}_{\text{tot}}^{2},S_{\text{tot}}^{z}]=0$ and that the generic Hamiltonian (1) commutes with $S_{\text{tot}}^{z}$. To verify that *H* commutes with ${\vec{S}}_{\text{tot}}^{2}$, one may show that each term in *H*, namely ${\vec{S}}_{i}\cdot {\vec{S}}_{j}(i\neq j)$, commutes with ${\vec{S}}_{\text{tot}}^{2}$. Evidently ${\vec{S}}_{l}^{2}$ commutes with ${\vec{S}}_{i}\cdot {\vec{S}}_{j}$ provided that *l* ≠ *i* and *l* ≠ *j*. If *l* equals *i*,

$$\begin{array}{cc} \left[{\vec{S}}_{l}^{2},{\vec{S}}_{l}\cdot {\vec{S}}_{j}\right] & =S_{l}^{a}[S_{l}^{a},S_{l}^{b}]S_{j}^{b}+[S_{l}^{a},S_{l}^{b}]S_{l}^{a}S_{j}^{b}\\ & \;\;=i\epsilon^{abc}(S_{l}^{a}S_{l}^{c}+S_{l}^{c}S_{l}^{a})S_{j}^{b}=0 \end{array}$$

due to the antisymmetric nature of the *ϵ* tensor. Other terms in *H* have the form ${\vec{S}}_{l}\cdot {\vec{S}}_{l^{\prime }}$. We write

(B.3) $$\begin{array}{cc} \sum_{l\neq l^{\prime }}{\vec{S}}_{l}\cdot & {\vec{S}}_{l^{\prime }}={\vec{S}}_{i}\cdot {\vec{S}}_{j}+{\vec{S}}_{j}\cdot {\vec{S}}_{i}+\sum_{l\neq l^{\prime }}^{\prime }{\vec{S}}_{l}\cdot {\vec{S}}_{l^{\prime }}\\ & \;\;+\sum_{l\neq i,l\neq j}{\vec{S}}_{l}\cdot \;({\vec{S}}_{i}+{\vec{S}}_{j})+\sum_{l^{\prime }\neq i,l^{\prime }\neq j}({\vec{S}}_{i}+{\vec{S}}_{j})\cdot {\vec{S}}_{l^{\prime }} \end{array}$$

where the indices *l* and *l*′ of $\sum_{l\neq l^{\prime }}^{\prime }$ do not overlap with *i* or *j*. Evidently, the first three terms of (B.3) commute with ${\vec{S}}_{i}\cdot {\vec{S}}_{j}$. However, ${\vec{S}}_{l}\cdot ({\vec{S}}_{i}+{\vec{S}}_{j})$ and $({\vec{S}}_{i}+{\vec{S}}_{j})\cdot {\vec{S}}_{l^{\prime }}$ both commute with ${\vec{S}}_{i}\cdot {\vec{S}}_{j}$. As an example, one may work out

(B.4) $$\begin{array}{cc} {}[{\vec{S}}_{l}\cdot ({\vec{S}}_{i} & +{\vec{S}}_{j}),{\vec{S}}_{i}\cdot {\vec{S}}_{j}]=S_{l}^{a}[S_{i}^{a}+S_{j}^{a},S_{i}^{b}S_{j}^{b}]\\ & \;=i\epsilon^{abc}S_{l}^{a}(S_{i}^{c}S_{j}^{b}+S_{j}^{b}S_{j}^{c})=0 \end{array}$$

due to the antisymmetric nature of the *ϵ* tensor. The relations above establish that ${\vec{S}}_{\text{tot}}^{2}$ commutes with ${\vec{S}}_{i}\cdot {\vec{S}}_{j}$. an arbitrary term in *H*, hence $[{\vec{S}}_{\text{tot}}^{2},H]=0$.

Next, the lowest energy state in the $S_{\text{tot}}^{z}=M$ subspace, henceforth called the M subspace, is shown to have $S_{\text{tot}}=M$. We expand somewhat the derivation of Lieb and Mattis (LM) here, hoping to make this appendix easier to read. We will largely follow the notations of LM whenever we can.

The basis set for the M subspace consists of tensor products all distinct eigenfunctions of the operators $S_{i}^{z}$ compatible with $S_{\text{tot}}^{z}=M$. Let *α* be the index that runs over each state in this set and ∣*χ*⟩ be the state in which the operator $S_{i}^{z}$ at site *i* has the lowest possible eigenvalue −*s_i_*. LM defined the phase of the states in the M subspace by

(B.5) $$|\phi_{\alpha }\rangle =C_{\alpha }(S_{1}^{+}{)}^{s_{1}+m_{1}}(S_{2}^{+}{)}^{s_{2}+m_{2}}\cdots (S_{N}^{+}{)}^{s_{N}+m_{N}}|\chi \rangle$$

With $\sum_{i=1}^{N}m_{i}=M$ and *C*_*α*_ being a *positive* normalization constant. Because these bases are eigenstates of $S_{i}^{z}$, one may separate the Hamiltonian into the diagonal part $H_{0}=2\sum J_{ij}S_{i}^{z}S_{j}^{z}$ and the nondiagonal part ${\tilde{H}}_{1}=\sum J_{ij}(S_{i}^{+}S_{j}^{-}+S_{i}^{-}S_{j}^{+})$. To simplify the nondiagonal part, LM introduced a canonical transformation by letting $S_{i(A)}^{x}\to -S_{i(A)}^{x},\;S_{i(A)}^{y}\to -S_{i(A)}^{y}$, and $S_{i(A)}^{z}\to S_{i(A)}^{z}$. This does not change *H*_0_ but ${\tilde{H}}_{1}$ is transformed to $H_{1}=-\left\{\sum |J_{ij}|S_{i}^{+}S_{j}^{-}+H.c.\right\}$ because the intra-sublattice coupling is negative (*J*_*i*(*A*),*j*(*A*)_ = −∣*J*_*i*(*A*),*j*(*A*)_∣ and *J*_*i*(*B*),*j*(*B*)_ = −∣*J*_*i*(*B*,*j*(*B*)_∣) and the inter-sublattice coupling is positive (*J*_*i*(*A*),*j*(*B*)_ = ∣*J*_*i*(*A*),*j*(*B*)_∣).

With the transformed Hamiltonian and the defined basis set, if one defines

(B.6) $$K_{\beta \alpha }=\langle \phi_{\beta }|H_{1}|\phi_{\alpha }\rangle ,$$

then *K*_*βα*_ < 0, i.e. *K*_*αβ*_ = −∣*K*_*αβ*_∣. To see this, we note that in order for *K*_*αβ*_ = 0, a term in *H*_1_ must be able to bring the state ∣*ϕ*_*α*_⟩ into ∣*ϕ_β_⟩*. If ∣*ϕ*_*α*_⟩ has at site *i* and *j* respectively the spin components *m_i_* + 1 and *m_j_* − 1 along the *z* direction while ∣*ϕ_β_*⟩ has at site *i* and *j* respectively the spin components *m_i_* and *m_j_* along the *z* direction, in *H*_1_ the term $-|J_{ij}|S_{i}^{-}S_{j}^{+}$ can bring the state ∣*ϕ*_*α*_⟩ to the state ∣*ϕ_β_*⟩. Now $S_{i}^{-}S_{i}^{+}=(S_{i}^{x}{)}^{2}+(S_{i}^{y}{)}^{2}-S_{i}^{z}={\vec{S}}_{i}^{2}-(S_{i}^{z}{)}^{2}-S_{i}^{z}$, implying that the matrix element *K*_*βα*_ ∝ −∣*J*_*ij*_∣ [*s_i_*(*s_i_* + 1) − *m_i_*(*m_i_* + 1)] = −∣*J_ij_*∣ [(*s_i_* – *m_i_*)(*s_i_* + *m_i_* + 1)]. Because-_s_i__ ⩽ *m_i_* ⩽ *s_i_*, *K*_*βα*_ ⩽ 0 is confirmed. (A bit of algebra shows that $K_{\beta \alpha }=-|J_{ij}|\sqrt{\left(s_{i}-m_{i})(s_{i}+m_{i}+1)(s_{j}+m_{j})(s_{j}-m_{j}+1\right)}$.) The realness of *K*_*βα*_ along with the fact that $K_{\beta \alpha }=K_{\alpha \beta }^{*}$ implies that *K*_*αβ*_ = *K*_*βα*_.

Denote the lowest energy state in the M subspace by *ψ* with energy $E_{M}$, we can expand *ψ* in terms of the complete set *ϕ*_*α*_ with amplitude *f*_*α*_

(B.7) $$|\psi \rangle =\sum_{\alpha }f_{\alpha }|\phi_{\alpha }\rangle .$$

Since ∣*ϕ*_*α*_⟩ is an eigenstate of *H*_0_, let us denote its eigenvalue by *e*_*α*_, meaning *H*_0_∣*ϕ*_*α*_⟩ = *e*_*α*_∣*ϕ*_*α*_). The Schrödinger equation thus can be written as

(B.8) $$\sum_{\beta }f_{\beta }(H_{0}+H_{1})|\phi_{\beta }\rangle =E_{M}\sum_{\beta }f_{\beta }|\phi_{\beta }\rangle .$$

Multiplying both sides by ⟨*ϕ*_*α*_∣ we obtain

(B.9) $$e_{\alpha }f_{\alpha }+\sum_{\beta }K_{\alpha \beta }f_{\beta }=E_{M}f_{\alpha }$$

or using *K_αβ_* = *K_βα_* = −∣*K_βα_*∣

(B.10) $$-\sum_{\beta }|K_{\beta \alpha }|f_{\beta }+e_{\alpha }f_{\alpha }=E_{M}f_{\alpha }.$$

The variational energy of any trial function exceeds $E_{M}$, unless it is also the lowest energy eigenfunction. LM mentioned that

(B.11) $$|\psi^{\prime }\rangle \equiv \sum_{\alpha }|f_{\alpha }|\;|\phi_{\alpha }\rangle$$

is a trial wave function with energy $E_{M}$ as well. We shall explicitly illustrate this point before carrying on with the analysis. Let

(B.12) $$E_{M}^{\prime }\equiv \frac{\langle \psi^{\prime }|H_{0}+H_{1}|\psi^{\prime }\rangle }{\langle \psi^{\prime }|\psi^{\prime }\rangle }$$

and we know $E_{M}^{\prime }⩾E_{M}$ by definition. (B.12) can be rewritten as

(B.13) $$E_{M}^{\prime }\left(\sum_{\alpha }|f_{\alpha }{|}^{2}\right)=-\sum_{\alpha ,\beta }|K_{\alpha \beta }||f_{\alpha }||f_{\beta }|+\sum_{\alpha }e_{\alpha }|f_{\alpha }{|}^{2}.$$

If we multiply both sides of (B.10) by $f_{\alpha }^{*}$ and sum over *α*, we obtain

(B.14) $$-\sum_{\alpha ,\beta }|K_{\beta \alpha }|f_{\alpha }^{*}f_{\beta }+\sum_{\alpha }e_{\alpha }|f_{\alpha }{|}^{2}=E_{M}\left(\sum_{\alpha }|f_{\alpha }{|}^{2}\right).$$

Hence

(B.15) $$\begin{array}{cc} (E_{M}^{\prime } & -E_{M})\left(\sum_{\alpha }|f_{\alpha }{|}^{2}\right)=-\sum_{\alpha ,\beta }|K_{\alpha \beta }|(|f_{\alpha }||f_{\beta }|-f_{\alpha }^{*}f_{\beta })\\ & =-\sum_{\alpha >\beta }|K_{\alpha \beta }|\;(2|f_{\alpha }|f_{\beta }|-f_{\alpha }^{*}f_{\beta }-f_{\alpha }f_{\beta }^{*})⩽0. \end{array}$$

This implies $E_{M}^{\prime }=E_{M}$ and the Schrödinger equation (B.10) satisfied by the eigenstate ∣**ψ*′⟩* reads

(B.16) $$-\sum_{\beta }|K_{\beta \alpha }|\;|f_{\beta }|+e_{\alpha }|f_{\alpha }|=E_{M}|f_{\alpha }|.$$

Furthermore, $(e_{\alpha }-E_{M})>0$ for all *α*. (Otherwise, one *ϕ*_*α*_ will have the lowest energy and be an eigenstate of *H*_0_ + *H*_1_; this is in general impossible.) Taking the absolute values on both sides of (B.10) and comparing with equation (B.16), one obtains

(B.17) $$|(\sum_{\beta }|K_{\beta \alpha }|f_{\beta })|=\sum_{\beta }|K_{\beta \alpha }|\;|f_{\beta }|.$$

This is possible only when *f_β_* ⩾ 0 for all *β* (or *f_β_* ⩽ 0 for all *β*). We may take the former without loss of generality. In fact, for the type of Hamiltonian we consider, we have *f_β_* > 0 for all *β*. For if one *f*_*α*_ = 0, then from (B.16) we have

(B.18) $$\sum_{\beta }|K_{\beta \alpha }|\;|f_{\beta }|=0$$

which is only possible if all ∣*ϕ_β_*⟩ that can be reached by applying *H*_1_ to ∣*ϕ*_*α*_⟩ have *f_β_* = 0 (zero amplitude). Then the states can be reached by applying *H*_1_ to those ∣*ϕ_β_*⟩ having *f_β_* = 0 must all have zero amplitude as well. Since for our case, successive application of *H*_1_ eventually covers every basis state in the M subspace, this implies that one must have *f_β_* > 0 for all *β*. Therefore, all amplitudes are positive and nonvanishing, hence $E_{M}$ is nondegenerate in the M subspace. This is because one cannot construct a state orthogonal to ∣*ψ*⟩ without some changes of signs. That is, any states orthogonal to ∣*ψ*⟩ must have amplitudes of opposite signs, hence not qualified as the lowest energy state (which we known must have positive and nonvanishing amplitude only).

We therefore arrive at the important conclusion of LM. For a system composed of two sublattices, any spin Hamiltonian describable by the general form (1) with nonpositive intrasublattice coupling (*J*_*i*(*A*),*j*(*A*)_ ⩽ 0 and *J*_*i*(*B*),*j*(*B*)_ ⩽ 0) and nonnegative inter-sublattice coupling (*J*_*i*(*A*),*j*(*B*)_ ⩾ 0), the lowest energy state belonging to the $S_{\text{tot}}^{z}=M$ (with M⩾0) subspace must have positive and nonvanishing projections along each basis vector satisfying $S_{\text{tot}}^{z}=M$. This implies that the lowest energy state of *H* and that of $H^{\prime }=({\vec{S}}_{\text{tot}}{)}^{2}-{\vec{S}}_{A}^{2}-{\vec{S}}_{B}^{2}$ (the soluable Hamiltonian introduced in the main text with ${\vec{S}}_{A}\equiv \sum_{i(A)}{\vec{S}}_{i(A)},{\vec{S}}_{B}\equiv \sum_{i(B)}{\vec{S}}_{i(B)}$, and ${\vec{S}}_{\text{tot}}=\sum_{i}{\vec{S}}_{i}={\vec{S}}_{A}+{\vec{S}}_{B}$) are not orthogonal to each other. Using the argument provided in the main text, we know these lowest energy states must have the same $S_{\text{tot}}=M$ in addition to having the same $S_{\text{tot}}^{z}=M$.
